# Cross-cultural adaptation and psychometric properties of the Arabic version of the Telerehabilitation Usability Questionnaire

**DOI:** 10.1080/07853890.2025.2525396

**Published:** 2025-06-30

**Authors:** Khalid H. Shebli, Fahad H. Alshehri, Yasir S. Alshehri, Yousef M. Alshehre, Rania N. Almeheyawi, Hosam Alzahrani

**Affiliations:** ^a^ Department of Physical Therapy, King Abdullah Hospital, Saudi Ministry of Health, Bisha, Saudi Arabia; ^b^ Department of Physical Therapy, College of Applied Medical Sciences, Taif University, Taif, Saudi Arabia; ^c^ Department of Physical Therapy, College of Medical Rehabilitation Sciences, Taibah University, Madinah, Saudi Arabia; ^d^ Department of Health Rehabilitation Sciences, Faculty of Applied Medical Sciences, University of Tabuk, Tabuk, Saudi Arabia

**Keywords:** Arabic, consistency, psychometric, reliability, questionnaire, telerehabilitation, usability

## Abstract

**Background:**

The objective of this study was to translate and cross‑culturally adapt the Telerehabilitation Usability Questionnaire (TUQ) into Arabic (TUQ-Arabic) and analyse the psychometric properties of the questionnaire.

**Patients and methods:**

Translation and cross-cultural adaptation have been conducted following international guidelines. A cohort of 270 Arabic-speaking participants completed the TUQ-Arabic. This study included participants who had utilised telerehabilitation services. Internal consistency was assessed with Cronbach’s alpha coefficient. The test–re-test reliability was performed on 69 participants using the intraclass correlation coefficient (ICC).

**Results:**

The cohort of the study comprised of 118 female (43.7%) and 152 male (56.3%) participants. Our findings indicate robust internal consistency in TUQ-Arabic subscales, exhibiting excellent Cronbach’s alpha values (>0.90, ranging from 0.902 to 0.940). The overall TUQ-Arabic score displayed excellent internal consistency (Cronbach’s alpha = 0.98). The test–re-test reliability of the total TUQ-Arabic score was excellent, with an ICC of 0.975 (95% CI 0.960–0.984, *p* < 0.001). Similarly, the test–re-test reliability of the TUQ-Arabic subscales ranged from good to excellent.

**Conclusion:**

The TUQ-Arabic is internally consistent and reliable, paralleling the original TUQ. Participants displayed favourable views on the telerehabilitation services, resulting in high satisfaction. The TUQ-Arabic is suitable for assessing the usability of remote telerehabilitation services in Saudi Arabia and populations globally.

## Introduction

The incorporation of telerehabilitation into healthcare systems has expanded rapidly, which has improved the accessibility and convenience of delivering rehabilitation services, specifically in remote or underserved regions [1]. Compared to traditional in-person therapy models, telerehabilitation has been demonstrated to be a cost-effective and practical approach for both patients and healthcare providers [2]. It also plays a critical role in increasing access to rehabilitation services, specifically in regions with limited healthcare infrastructure, by facilitating remote communication between patients and providers. The COVID-19 ­pandemic further accelerated the implementation of telerehabilitation, emphasising its potential to overcome constraints including geographical barriers, resource shortage, and limited mobility [3]. As healthcare providers increasingly integrate telerehabilitation into their practice [4], the usability of these systems becomes a crucial aspect impacting their effectiveness and acceptance.

Despite the advancement of telerehabilitation services globally, the usability of these technologies has primarily been explored using instruments developed and validated in Western contexts. Existing instruments comprehensively explore crucial factors including usefulness, ease of use, interaction quality, interface quality, reliability, and satisfaction and future use. The Telerehabilitation Usability Questionnaire (TUQ) is one such tool that has been developed to explore telerehabilitation systems from both provider and patient perspectives [5]. Recognising the significance of cultural and linguistic relevance, various studies have translated the original TUQ into Danish [6], Brazilian Portuguese [7], and Turkish [8], thus enabling its application in various populations. However, cultural, and linguistic variations of the TUQ necessitate further adaptation and validation to ensure their applicability and reliability in Arabic-speaking populations.

In Arabic-speaking countries, particularly Saudi Arabia, telerehabilitation has gained popularity in recent years, driven by technological developments and increasing demand for accessible healthcare solutions. However, research on the usability and usefulness of these systems in the Arabic-speaking countries context remains scarce. The lack of validated instruments for examining telerehabilitation usability in this context poses a major barrier to understanding user demands and improving system design. To address this gap, the current study aimed to translate and cross‑culturally adapt the TUQ into Arabic and assess its psychometric properties.

## Materials and methods

### Study design and setting

This methodological research study received ethical approval from the Scientific Research Ethics Committee at Taif University in Saudi Arabia (reference number: 44-081). Participants provided electronic informed consent before completing the online questionnaire. The electronic consent form was presented on the first page of the online questionnaire. The form involved information about the study’s purpose, procedures, confidentiality, and voluntary nature of participation. Participants provided informed consent by clicking ‘Next’ and proceeding to complete the questionnaire, indicating their agreement to participate. This study adheres to the Declaration of Helsinki.

### Translation and adaptation process of the TUQ

Permission to translate and cross‑culturally adapt the TUQ was obtained from the developers of the questionnaire. The original English version of the questionnaire was translated and adapted to Arabic following international guidelines [9,10].

The process of translating and adapting the questionnaire involved various phases, each designed to ensure that the final version was appropriate and effective for the target population. In the first phase, two bilingual professional translators who were native Arabic speakers produced independent forward translations of the questionnaire. Then, the two translated versions produced in this phase were reviewed by two physical therapists who made cultural adaptations to ensure the questionnaire was appropriate for Arabic-speaking patients. The second phase involved synthesising the two independent translations from English to Arabic in one integrated Arabic version. The synthesis process involved comparing the two translations and identifying their differences and similarities. The translators then worked together to resolve discrepancies and produced one version that combined the best aspects of both translations. In the third phase, two independent bilingual native English-speaking translators conducted a backward translation from Arabic to English to ensure that the Arabic version was sustained with the original questionnaire. These translators had no medical backgrounds to avoid information bias and allow unexpected meanings of items in the translated questionnaire to emerge. In the fourth phase, the initial English survey was compared with the backward-translated adaptations by a committee of specialists working within the field of English basics and translation. In the fifth phase, a pilot post-translation was conducted with 13 participants randomly selected from an Arabic-speaking patient group.

### Participants

This study recruited adults who possess a satisfactory level of proficiency in the Arabic language and had used TR services. Participants were recruited through rehabilitation clinics, and eligibility criteria were evaluated to confirm both their proficiency and previous engagement with telerehabilitation. This study excluded participants with past histories of mental disorders and/or patients with conditions that may impact their understanding. The current study employed a non-random convenience sampling method. The online questionnaire was first shared with practicing physiotherapists and rehabilitation physicians working in clinics and departments across the different regions of Saudi Arabia. These practitioners then forwarded the survey link to eligible patients in their clinics. At the beginning of the questionnaire, all respondents answered screening questions to confirm their eligibility to participate in the study. This multi-stage, clinician-mediated sampling ensured wide geographic coverage and that only eligible patients completed the survey.

### Sample size estimation

The sample size was determined in accordance with existent recommendations, which state that there should be at least 10 respondents for each question in a scale [11]. The TUQ consists of 21 items. Therefore, this study required at least 210 participants to ensure an adequate sample size for measuring psychometric properties of the translated TUQ.

### Data collection

The questionnaire was administered using Google Forms and distributed to eligible participants through various electronic platforms, such as emails and Short Message Service. Before completing the TUQ-Arabic version, participants were asked to provide information about their sociodemographic characteristics, including age, gender, marital status, and level of education.

The TUQ consists of 21 items and evaluates individuals’ satisfaction with and usability of TR services [5]. The questionnaire assesses the following domains: usefulness (3 items), ease of use and learnability (3 items), interface quality (4 items), interaction quality (4 items), reliability (3 items), and satisfaction and future use (4 items). A 7-point Likert scale was used in the TUQ evaluation (strongly disagree [1], disagree [2], somewhat disagree [3], neutral [4], somewhat agree [5], agree [6], and strongly agree [7]). The total score was calculated by summing the 21 items (21–147). A higher total score indicates greater usability.

### Statistical analysis

All analyses were conducted utilizing SPSS software (version 26.0, IBM Corp., Armonk, NY, USA). Mean and standard deviation were provided for the quantitative information. Percentiles were displayed for the qualitative information. The internal consistency of the TUQ-Arabic subscales and the total score were assessed utilizing Cronbach’s alpha test. Cronbach’s alpha value between 0.70 and 0.95 indicates adequate internal consistency [12]. The test–re-test reliability of the TUQ-Arabic was evaluated by two-way random intraclass correlation coefficients (ICCs) for absolute agreement with their corresponding 95% confidence intervals (95% CIs) [12]. The reliability was considered ‘excellent’ if ICC ≥0.75; ‘good’ if 0.40 ≤ ICC < 0.75, and ‘poor’ if ICC < 0.40. The standard error of measurement (SEM) was computed utilizing the formula: $\text{SEM}=\text{SD}\times \sqrt{1-\text{ICC}}$, where the standard deviation (SD) is the pooled standard deviation. The smallest detectable change (SDC) was computed utilizing the formula: $\text{SDC}=1.96\times \sqrt{2}\times \text{SEM}$ [12].

## Results

### Translation and cross-cultural adaptation of the TUQ

The TUQ questionnaire was translated from English to Arabic without major problems. The back translation of the questionnaire was also conducted without any major linguistic or grammatical problems. The results of the pre-test phase showed that the items of the TUQ-Arabic were understandable in terms of language and cultural concepts. Therefore, no specific changes were made after the pre-test phase.

### Participant characteristics

A total of 270 participants completed the TUQ-Arabic. Of these participants, 118 were female (43.7%) and 152 were male (56.3%). Participants between the ages of 21–30 years old represented the highest percentage (34.1%) in the sample. Nearly two-thirds of the participants used TR services *via* the Ministry of Health hospitals in Saudi Arabia, followed by 17.4% of the participants *via* the Ministry of Health primary healthcare centres. Approximately 96% of the participants answered ‘yes’ when asked if they have computer proficiency. For the question related to how many TR appointments they had attended, 47.4% of the participants reported 2–5 appointments, followed by only one appointment (39.3%). When asked about the status of their general health, most participants reported excellent health. A summary of the participants’ characteristics is presented in Table 1.

**Table 1.** Demographics and distribution of study participants.

	Sample size *n* = 270
Sex, *n* (%)	
Female	118 (43.7)
Male	152 (56.3)
Age, years, *n* (%)	
18–20	42 (15.6)
21–30	92 (34.1)
31–40	82 (30.4)
41–50	38 (14.1)
51–60	11 (4.1)
>60	5 (1.9)
Highest level of education, *n* (%)	
High school or diploma	85 (31.5)
Bachelor’s degree	165 (61.1)
Postgraduate degree	20 (7.4)
Region in Saudi Arabia, *n* (%)	
Central	114 (42.2)
Western	75 (27.8)
Eastern	16 (5.9)
Southern	58 (21.5)
Northern	7 (2.6)
Where did you receive the telehealth service? *n* (%)	
MOH primary healthcare centre	47 (17.4)
Private sectors (centres or hospitals)	11 (4.1)
MOH hospitals	183 (67.8)
University hospitals	14 (5.2)
Military hospitals	12 (4.4)
Other	3 (1.2)
Computer proficiency, *n* (%)	
Yes	259 (95.9)
No	11 (4.1)
How many telerehabilitation appointments have you attended? *n* (%)	
<1	2 (0.7)
Only 1	106 (39.3)
2–5	128 (47.4)
6–9	27 (10.0)
>9	7 (2.6)
General health status, *n* (%)	
Bad	1 (0.4)
Good	7 (2.6)
Very good	30 (11.1)
Excellent	232 (85.9)
TUQ-Arabic, M ± SD	
Usefulness	18.85 ± 3.65
Ease of use and learnability	18.73 ± 3.58
Interface quality	24.96 ± 4.85
Interaction quality	25.10 ± 4.68
Reliability	18.29 ± 4.24
Satisfaction and future use	24.94 ± 5.22
Total score	**130.88 ± 24.38**

MOH, The Saudi Ministry of Health; TUQ-Arabic, Arabic version of the Telerehabilitation Usability Questionnaire; M, mean; SD, standard deviation.

### Internal consistency

The internal consistency of both the TUQ-Arabic subscales and the TUQ-Arabic total score were excellent (respectively, Cronbach’s alpha = 0.98 and >0.90, ranging from 0.902 to 0.940). The results of the internal consistency are shown in Table 2.

**Table 2.** Internal consistency of the TUQ-Arabic subscales and total score.

	Cronbach’s alpha
Usefulness	0.90
Ease of use and learnability	0.91
Interface quality	0.94
Interaction quality	0.92
Reliability	0.90
Satisfaction and future use	0.94
Total score	0.98

### Test–re-test reliability

In total, 69 participants completed the TUQ-Arabic twice (Table 3). Participants were asked to complete the TUQ-Arabic test again within 4–7 days of completing the questionnaire for the first time. The results showed that the test–re-test reliability of the TUQ-Arabic subscales was good to excellent, with ICCs _2, 1_ ranging from 0.578 to 1. The test–re-test reliability of the TUQ-Arabic total score was excellent, with an ICC _2, 1_ of 0.975 (95% CI 0.960–0.984, *p* < 0.001). The SEM of the TUQ-Arabic subscales ranged from 0 to 0.933, whereas the SDC ranged from 0 to 2.59. The SEM and SDC of the TUQ-Arabic total scores were 1.358 and 3.76, respectively. A summary of the reliability results is presented in Table 4.

**Table 3.** Demographic and characteristics of participants who completed the questionnaire twice for test–retest reliability analysis (*n* = 69).

	Sample size *n* = 69
Sex, *n* (%)	
Female	31 (44.9)
Male	38 (55.1)
Age, years, *n* (%)	
18–20	9 (13.0)
21–30	22 (31.9)
31–40	23 (33.3)
41–50	13 (18.8)
51–60	1 (1.5)
>60	1 (1.5)
Highest level of education, *n* (%)	
High school or diploma	17 (24.6)
Bachelor’s degree	50 (72.5)
Postgraduate degree	2 (2.9)
Region in Saudi Arabia, *n* (%)	
Central	43 (62.3)
Western	19 (27.5)
Southern	7 (10.1)
Where did you receive the telehealth service? *n* (%)	
MOH primary healthcare centre	10 (14.5)
Private sectors (centres or hospitals)	1 (1.5)
MOH hospitals	58 (84.0)
Computer proficiency, *n* (%)	
Yes	68 (98.5)
No	1 (1.5)
How many telerehabilitation appointments have you attended? *n* (%)	
Only 1	25 (36.2)
2–5	31 (44.9)
6–9	13 (18.8)
General health status, *n* (%)	
Good	1 (1.5)
Very good	9 (13.0)
Excellent	59 (85.5)

**Table 4.** Test–re-test reliability of the TUQ-Arabic subscales and total score.

	Participants *n* = 69 M ± SD		ICC (95% CI)	SEM	SDC
	1st session	2nd session			
Usefulness	20.13 ± 1.66	20.24 ± 1.17	0.578	0.933	2.59
Ease of use and learnability	20.26 ± 1.40	20.19 ± 1.39	0.908	0.423	1.17
Interface quality	26.85 ± 2.15	26.83 ± 2.15	0.996	0.136	0.38
Interaction quality	26.52 ± 2.97	26.54 ± 2.83	0.979	0.420	1.17
Reliability	19.89 ± 2.10	19.94 ± 2.03	0.985	0.253	0.70
Satisfaction and future use	27.13 ± 1.64	27.13 ± 1.64	1	0	0
Total score	140.78 ± 9.09	140.87 ± 8.05	0.975	1.358	3.76

ICC, intraclass correlation coefficient; SEM, standard error of measurement; SDC, smallest detectable change; M, mean; SD, standard deviation.

## Discussion

Our research demonstrated that the TUQ-Arabic exhibited high internal consistency and reliability among Arabic-speaking patients who received TR services. The Arabic version of the scale showed adequate psychometric properties that were comparable to those of the original English version and the translated versions of the scale [5–8].

The internal consistency of the TUQ-Arabic total score was excellent (Cronbach’s alpha = 0.98). Further, the internal consistency of the TUQ-Arabic subscales was excellent (Cronbach’s alpha >0.90, ranging from 0.902–0.940). The results of this study agree with the original English version of the TUQ [5], which demonstrated a Cronbach’s alpha of 0.8. Furthermore, Cronbach’s alpha obtained in this study are comparable to those reported in other studies of the Turkish (Cronbach’s alpha >0.90) [8], Danish (Cronbach’s alpha = 0.857) [6], and Brazilian (Cronbach’s alpha >0.90) [7] versions of the scale. Likewise, the test–re-test reliability of the TUQ-Arabic total score was excellent (ICC = 0.975), which was higher than that obtained in the Turkish study (ICC = 0.872) [8]. However, it is important to note that the test–re-test reliability of the English version of the instrument was not explored in the original study [5].

The findings regarding the SEM and the SDC for the TUQ-Arabic total scores provide important insights into the instrument’s reliability and sensitivity. An SEM of 1.358 signifies that the TUQ-Arabic has a low level of measurement error, suggesting that the questionnaire yields consistent results across repeated administrations in a stable population [13]. This level of precision indicates the strong capability of the instrument to reliably explore telerehabilitation usability. Moreover, an SDC of 3.76 indicates that any change in the instrument total score exceeding this value can be considered a true change beyond measurement error [14], suggesting that the TUQ-Arabic is sufficiently sensitive to determine meaningful variations in usability perceptions over time or after interventions. Together, these results show that the TUQ-Arabic is a reliable instrument for assessing the usability of telerehabilitation systems among Arabic-speaking individuals, enhancing its applicability in both research settings and clinical practice.

The results of the study suggest that there may be differences in the usability and acceptance of TR services among different regions of Saudi Arabia. The surveyed participants were from different geographical regions of Saudi Arabia, with most (42.2%) being from the Central region, followed by the Western region (27.8%). The distribution of participants in our study might be due to several factors. The Central and Western regions of Saudi Arabia are more urbanised and have a higher level of access to healthcare services, including TR services, compared to the Northern and Southern regions, which are more rural and less developed [15]. Additionally, the Central region is the most populous in the country and includes the capital city of Riyadh, which is a major hub for healthcare services and research [16].

The participant characteristics offer valuable context for assessing the psychometric properties of the Arabic-TUQ version. Many participants frequently utilised telerehabilitation services, with many attending multiple appointments, reflecting their trust in and satisfaction with these services. This high attendance rate may also suggest that the telerehabilitation services were accessible and aligned with patient needs, thereby supporting the validity of responses in the usability assessment. Notably, most participants accessed telerehabilitation services through MOH facilities, with much lower utilisation reported for university, private sector, and military healthcare providers. This imbalance indicates the dominant role of MOH in delivering telerehabilitation services in Saudi Arabia and may also impact the generalizability of the study results to other service providers. Moreover, the limited involvement of the private sector and social media platforms in promoting TR services underscores a potential gap in outreach that limits patient awareness. Age-related differences were also observed, with younger adults (aged 21–40 years) comprising the largest group of participants, which is consistent with previous research indicating that younger populations are more likely to engage with technology-based healthcare solutions [17–19].

Some limitations need to be considered when interpreting our findings. First, telerehabilitation encompasses a variety of users, technologies, and organisational structures. Thus, to assure that the TUQ-Arabic questionnaire is applicable to all available telerehabilitation solutions, a pertinent next step would be to evaluate the TUQ-Arabic questionnaire on various telerehabilitation solutions with diverse user groups. Another limitation is that this study used a convenience sample which may impact the generalizability of the TUQ-Arabic questionnaire to other samples. However, this type of sample selection is the most used in studies evaluating the psychometric properties of instruments such as the TUQ.

## Conclusion

The TUQ-Arabic showed high internal consistency and reliability among Arabic-speaking individuals who received TR services. The questionnaire can be used to evaluate the usability of remote telerehabilitation services in Saudi Arabia and possibly in other Arabic-speaking countries.

## Data Availability

The data supporting the findings of this study are available from the corresponding author upon reasonable request.
